# A qualitative description of barriers to visual rehabilitation experienced by stroke survivors with visual impairment in Alberta, Canada

**DOI:** 10.1186/s12913-023-09064-9

**Published:** 2023-01-20

**Authors:** Kiran Pohar Manhas, Katelyn Brehon, Jennis Jiang, Karim F. Damji, Fiona Costello

**Affiliations:** 1 Neurosciences, Rehabilitation and Vision, Strategic Clinical NetworkAlberta Health Services, 10301 Southport Lane SW, Calgary, AB T2W 1S7 Canada; 2 Department of Community Health Sciences, Cumming School of Medicine, University of Calgary, Calgary, AB Canada; 3 Faculty of Nursing, College of Health Sciences, University of Alberta, Edmonton, AB Canada; 4 Department of Physical Therapy, Faculty of Rehabilitation Medicine, University of Alberta, Edmonton, AB Canada; 5 Department of Ophthalmology and Visual Sciences, Faculty of Medicine and Dentistry, College of Health Sciences, University of Alberta, Edmonton, AB Canada; 6 Department of Ophthalmology and Visual Sciences, Aga Khan University, Karachi, Pakistan; 7 Departments of Clinical Neurosciences and Surgery, Cumming School of Medicine, University of Calgary, Calgary, AB Canada

**Keywords:** Stroke, vision loss, qualitative, patient experience, provider experience

## Abstract

**Background:**

Post-stroke visual impairment (VI) is a common but under-recognized care challenge. Common manifestations of post-stroke VI include: diplopia, homonymous hemianopia, oscillopsia secondary to nystagmus, and visual inattention or neglect. In acute care settings, post-stroke VI recognition and treatment are often sub-optimal as emphasis is placed on survival. Stroke survivors with VI often face inconsistencies when accessing care out of hospital because variable availability and subsidization of visual rehabilitation. We sought to identify gaps in care experienced by stroke survivors with VI from stroke survivors’ and care providers’ perspectives.

**Methods:**

We conducted a qualitative description study across 12 care sites in Alberta, Canada, using semi-structured interviews. Survivor interviews focused on the health system experience. Provider interviews discussed approaches to care, perceived gaps, and current resources. Interviews were audio-recorded and transcribed. Iterative content analysis was completed using NVivo 12. We promoted rigour through an audit trail, open-ended questions, thick description, and collaborative coding.

**Results:**

We completed 50 interviews: 19 survivor interviews and 31 provider interviews. The majority of survivors were male (n = 14) and recruited from community settings (n = 16). Providers varied in profession and location within the care continuum. Two key themes emerged from the provider and survivor interviews pertaining to (a) facets of visual rehabilitation (sub-themes: access, resources, and multidisciplinary professional interaction); and (b) functioning with post-stroke VI (sub-themes: early experiences post-stroke and living with VI in the real world).

**Conclusions:**

The visual rehabilitation model needs to be optimized to ensure transparent inter-disciplinary communication and efficient referral pathways. Future research will focus on evaluating the effectiveness of post-stroke care from multiple perspectives in Alberta.

## Introduction

Stroke is the second leading cause of death worldwide [1]. In Canada, stroke represents the leading cause of adult disability and the third leading cause of death [2]. Globally, the burden of stroke continues to increase due to population growth, aging, and increased prevalence of modifiable risk factors [1].

Post-stroke visual impairment (VI) is a common, but under-recognized, care challenge [3, 4]. Common manifestations of post-stroke VI include: diplopia, homonymous hemianopia, oscillopsia secondary to nystagmus, and visual inattention or neglect [5]. Affected individuals frequently report decreased quality of life, loss of independence, depression, and social isolation [6–8]. Post-stroke VI negatively impacts recovery from other stroke sequelae [6–8], and addressing it during rehabilitation is necessary. Visual rehabilitation may include modification to environments, training in basic and instrumental activities of daily living (ADLs and IADLs), use of optical devices, and online education [9, 10]. In acute care settings, post-stroke VI recognition and treatment are often sub-optimal as emphasis is placed on survival, since 12.3% of strokes are fatal within the first 30 days following hospital admission [11]. Challenges may arise when patients have neurological or cognitive deficits including hemi-neglect and aphasia, which impede articulation, thus masking VI manifestations.

Post-stroke visual rehabilitation has been shown to improve quality of life outcomes. Individuals with visual field deficits may visualize objects in the preserved field of view with the aid of mirrors or prisms [3]. Scanning techniques can facilitate item location, reading, and driving post-stroke [5]. Studies suggest that access to visual rehabilitation post-stroke is inconsistent. American ophthalmologists (*n* = 143) rarely refer stroke patients with unilateral or bilateral VI for rehabilitation [12]. Among 459 Canadian optometrists surveyed, merely 10.7% indicated they would manage patients with VI requiring specialized devices [13]. Only 54–63% (*n* = 108) of American school-based occupational therapists reported feeling comfortable providing visual screening and rehabilitation for VI [14]. Unfortunately, even when post-stroke visual rehabilitation resources are available, they may be under-utilized [15, 16].

In Canada, stroke survivors with VI face inconsistencies when accessing care because levels of subsidization of visual rehabilitation vary provincially [17]. In Quebec, visual rehabilitation is publicly funded whereas Albertans often require private insurance to subsidize costs. Similar variations can be detected globally. The objective of this study was to identify gaps in care experienced by stroke survivors with VI in Alberta by describing (a) survivors’ experience of VI diagnosis and management across the care continuum, including perceived barriers and facilitators; and (b) provider perceptions of health services delivery and interprofessional collaboration in these settings.

## Methods

### Study design

This qualitative description study was conducted at 12 sites in Alberta, from August 2020 to February 2021. Qualitative description seeks insights from informants, emphasizing the characteristics of poorly-understood phenomena [18]. The tenants of qualitative description include: (a) a naturalistic, (b) purposive sample; (c) semi-structured interviews; and, (d) content analysis [18, 19]. This study received approval from the University of Calgary’s Conjoint Health Research Ethics Board. All study participants provided informed consent.

### Study population

Study participants represented the care continuum, including: acute stroke units, tertiary in-patient rehabilitative units, and community-based settings (including vision and rehabilitation clinics).

#### Inclusion criteria

Adult stroke survivors (aged 18 years and older) who were able to read and understand English and had visited a participating site within the last three years were recruited. Care providers involved in the management of survivors were eligible to participate (e.g. allied health, neurologists, nurses, ophthalmologists, optometrists).

#### Exclusion criteria

Survivor exclusion criteria included lacking the cognitive or communicative capacity to provide meaningful informed consent. There were no exclusion criteria for providers.

### Recruitment strategies

Purposive sampling towards maximum variation directed recruitment. We recruited until saturation across study sites for geographical representation of survivors and providers as well as diversity of providers’ professional designations. We did not recruit survivors from acute settings due to COVID-pandemic-related access limitations. Instead, we sought survivors with acute inpatient experiences. For study feasibility and site diversity, we sought contact from four to five survivors and four to five providers, per site.

Recruitment strategies were co-designed with site management. At each site, provider recruitment involved team-wide overview presentations followed by an emailed invitation to teams (sent by managers not researchers). Providers who were interested in participated contacted researchers, who then had 1:1 conversations to obtain informed written consent. At each site, survivor recruitment was led by a site-specific staff liaison. The staff liaisons identified and approached eligible stroke survivors to discuss study participation. They obtained permission to share contact information with researchers, who then followed up with study participants directly. Follow-up with survivors involved informed consent discussions as well as organizing the interviews at a date and time convenient for the survivors.

### Data collection

Interviews were conducted by two experienced interviewers trained in qualitative methods. Interviews were 1:1 and were by phone or videoconference (Zoom or Skype for Business). We used a semi-structured question guide. Survivor interviews focused on the experience of the health system from initial stroke symptoms to the time of the interview. Probing questions were used to elicit perceptions on the nature, timeliness, and appropriateness of VI diagnosis and management. Provider questions discussed approaches to caring for survivors in their current setting, perceived gaps in the system, and current collaborative practices with other professions in VI care. Interviews were audio recorded and confidentially transcribed verbatim. Field notes were made during each interview.

### Data analysis

Qualitative content analysis directed analysis of transcripts and field notes, using NVivo 12. An initial coding framework was created using methods described by Patton [20]. The coding framework was created iteratively by three team members. Each team member initially coded 5 (26%) survivor, and 5 (16%) provider, transcripts with some overlap. Survivor and provider interviews were analyzed separately. After initial coding, the team integrated their respective coding into an over-arching coding framework. Survivor and provider coding frameworks were collapsed due to similarities in emergent codes. Two researchers applied the combined framework to the remaining transcripts. The team was open and receptive to emergent codes throughout this process.

We promoted qualitative rigour by using an audit trail of decisions for accountability, employing open-ended questions to prioritize participant voices, ensuring thick description for fidelity of participant voice, and implementing collaborative coding to expose biases during analysis.

## Results

Fifty interviews were completed. Thirty-one providers participated (15–54 min) who varied in profession (occupational therapy, physical therapy, speech language pathology, therapy assistants, neurology, ophthalmology, optometry, optics, and orthoptics) and location along the care continuum. Most provider-participants were from community care settings (*n* = 20, 64.5%). Nineteen stroke survivors participated (10–75 min), with representation from urban and rural areas. Most survivor-participants were male (*n* = 14, 73.7%) and were recruited from community care settings (*n* = 16, 84.2%).

**Fig. 1:**
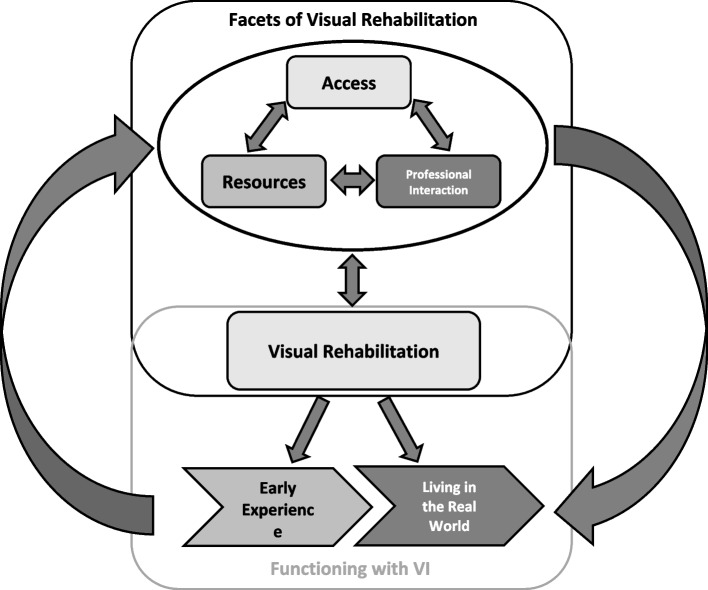
Thematic framework highlighting the relationships between facets of visual rehabilitation and functioning with VI. There is a bidirectional arrow between access, resources, and professionals and visual rehabilitation since the services provided to survivors can also affect future access to professionals and various other resources**.** The curved arrows on either side of the framework show how the early stroke experiences can influence barriers and facilitators to visual rehabilitation such as better or worse access, resources, and professionals. Facets of visual rehabilitation can also influence how survivors are cared for, and function, later on when they are living in the real world

Two key themes emerged from the provider and survivor interviews: (a) the facets of visual rehabilitation; and (b) functioning with post-stroke VI (Fig. 1). In theme (a), providers and survivors described three sub-themes relating to barriers and facilitators to visual rehabilitation: access, resources, and multidisciplinary professional interaction. In theme (b), providers and survivors spoke of their respective roles and experiences around advancing post-stroke VI. Sub-themes included early experiences post-stroke and living with VI in the real world. Themes and sub-themes were not mutually exclusive. Table 1 includes a description of each theme and sub-theme.

**Table 1:** Description of key themes and sub-themes

Theme/Sub-Theme	Meaning in Framework
Facets of Visual Rehabilitation	Factors that improved or hindered visual rehabilitation care received by stroke survivors as well as how they may be improved
Access	Factors affecting whether or not visual rehabilitation was available to stroke survivors. Examples include financial considerations such as the costs of rehabilitation programs as well as those pertaining to driving, travel, and transportation; timing; or mode of delivery (virtual or in-person)
Resources	Factors related to providers familiarity, and usage of, visual rehabilitation as well as the availability of equipment, devices, or certain supports like Early Supported Discharge teams
Multidisciplinary Professional Interaction	Factors related to inter- and intra-professional/team communication
Functioning with VI	Factors related to early experiences post-stroke and how the transition to living in the real-world affects stroke survivors’ ability to function with their VI
Early Experience	Factors related to stroke survivors’ acute care experiences including screening and diagnosis activities
Living in the Real World	Factors related to management and rehabilitation activities; compensation versus full recovery; as well as driving and transportation

### Theme A: Facets of visual rehabilitation

Providers spoke about gaps in the health system and opportunities for improvement. Survivors spoke about how access, resources, and multidisciplinary professional interaction improved or hindered their visual rehabilitation care.

#### Access

Survivors and providers noted the lack of provincial public funding programs. Visual rehabilitation and devices represent out-of-pocket costs for survivors. Survivors found injustice in this lack of subsidization when devices for other stroke sequelae, like impaired mobility, were often covered. Optometrists wanted to provide publicly-funded vision rehabilitation as part of their regular basket of services; however, current infrastructure costs exceed public compensation leading to limited community-based settings for vision rehabilitation. While assistive funding programs for stroke survivors exist, providers varied on their knowledge on referral processes and program access requirements. Capacity to cover high costs determined survivors’ ability to travel for vision rehabilitation.

Providers noted that survivors were often distressed when asked to surrender their driver’s licenses due to post-stroke VI. Losing driving rights left survivors often reliant on family members, taking public transit, or arranging alternative transportation. Dependence on others brought additional challenges including scheduling conflicts and travel expenses. In rural contexts, public transportation was less readily available. Providers worried most about survivors with the trifecta of living alone, living in rural settings, and having lost driving privileges. Significant wait times (1–6 months) exacerbated access barriers.

“when I went to the post-stroke clinic, … it was supposed to be … six to eight weeks after the stroke, but it was like five months” (male survivor, community)

“the lady at the [hospital] told me it’ll probably be a month before I get in.. because she’s busy” (female survivor, tertiary inpatient rehabilitation)

*“Perhaps … providing … a bit more … marketability or exposure to low vision services. I’m always shocked meeting a person who’s been legally blind or had suffered a stroke and that may have been years ago and they never even … had the opportunity to access any type of service or program … that might help to keep them independent” (female provider, community)*

#### Resources

Resource scarcity was another recognized barrier. In rural areas, providers noted that community resources had closed, thus decreasing access to visual rehabilitation for survivors. An example was the recent, Fall 2019, closing of the non-metropolitan offices of a community service organization offering vision rehabilitation provincially. To access resources where offices had closed, survivors had to travel to larger cities or wait until providers visited rural settings. These challenges increased the cost and wait times for access to rehabilitation.

At certain community care sites, survivors were availed of technologies like the DynaVision. Survivors who used this type of equipment at least once wished that they could use it regularly during their rehabilitation journey. Survivors felt that in tertiary rehabilitation centres, there lacked sufficient vision-related exercises compared to the number of physical exercises available to improve their other stroke sequelae.

Not all sites and areas of the province were equipped with a stroke Early Supported Discharge (ESD) team, which provided multidisciplinary, acute-level care in the patient’s home upon early discharge from hospital. Geographical limits dictated client eligibility. Some ESD teams stretched service boundaries to promote access, but other ESD teams were perceived as less adaptive to patient needs, leaving some survivors unable to access in-home care. Given that most stroke survivors with post-stroke VI are unable to drive, this geographical limitation posed a significant challenge to accessing rehabilitation. ESD-eligible survivors discussed how it was a useful, convenient service that allowed them to recover at home.

*“…there’s lots of anxiety with the double vision, lots of impairment functionally so more often they’re struggling with walking, doing their routine IADLs like going for groceries so … with that particular patient, he did report a lot more satisfaction with coming for [large-board] treatment [in-person], he felt like it was helping him” (female provider, tertiary)*

*“[it] was really great … and … especially too, [ESD] helping out with not just me but also my spouse… and giving her the information too, and that … was also great because a lot of times too … your spouse is the one that needs to know what is going on as well because a lot of times they feel like they’re left in the dark, right?” (male survivor, community)*

#### Multidisciplinary Professional Interaction

Community care teams tended to have interdisciplinary or transdisciplinary approaches to visual rehabilitation post-stroke. ESD teams discussed how they developed a rehabilitation plan as a team and how multiple professions could do tasks, like the initial screen for post-stroke VI.

Communication and collaboration within health care teams was inconsistently perceived as strong. Some providers described interprofessional communication between teams as requiring improvement, particularly between outpatient ophthalmology or optometry clinics and community care. Better communication on specific diagnoses from the ophthalmologist or optometrist could help allied health professionals tailor rehabilitation strategies. Allied health professionals were unsure of where or when to refer for ophthalmologic or optometric services, further contributing to communication barriers.

Some survivors described how lack of communication between providers contributed to referral delays. Provider-to-survivor communication was considered inadequate; all survivors experienced some lack of information about post-stroke VI throughout their care journey, especially during the acute-care phase. They discussed how isolated they felt when providers recommended a “wait-and-see” approach to their VI, which was coupled with limited resources or support. Some of these “wait-and-see” survivors resorted to researching visual exercises online; one survivor designed their own constraint-induced therapy glasses. Information paucity seemed to improve as survivors progressed through the care continuum. Tertiary and community care providers were able to more readily address questions related to visual rehabilitation and living with post-stroke VI in the real-world; however, some survivors had to self-advocate and query about visual rehabilitation for it to be discussed.

*“I think there are many players and at the end of the day we all … contribute from a slightly different perspective so I think that for the vision loss that’s stroke-related in particular … it would be difficult to streamline that … to one provider because it’s so unique to each context and every stroke is so unique in terms of its constellation of impairments. … I think that maintaining the interdisciplinary approach is really helpful” (female provider, community)*

*“Well I think we’re doing better in opening … the communication gap. We … kind of go forward and then we’ll just plateau for a while so although we’re working on it, I still feel there’s gaps in how we communicate to each other … and provide each other with information…” (female provider, community)*

*“… the only care to do with my vision … of any kind [that I received], [was] I… requested from my neurologist to see … a neuro-ophthalmologist … to see if there’s a special surgery or something for me, which there wasn’t. But I … have been seen by him and … learned of … prism glasses.” (male survivor, community)*

### Theme B: Functioning with VI

Functioning with post-stroke VI relates to early experiences and living in the real-world. Providers spoke about their visual rehabilitation roles related to function and where in the care continuum they encountered most survivors. Survivors spoke about their experiences with functioning with VI shortly post-stroke and how their functioning progressed as they transitioned home and into the community.

#### Early experiences

In the acute care setting, vision needs were seen as secondary concerns to stroke cause and secondary stroke prevention. Acute care providers approached vision needs mainly in terms of screening and diagnosis. Occasionally, providers from in-patient tertiary care offered some vision care. Yet, if the survivor was dealing with other stroke sequelae, vision was not the focus of their rehabilitation plan. If VI was the only symptom resulting from stroke, these survivors were generally discharged home sooner than survivors faced with additional stroke sequelae.

*“I think at that point in the more severely involved ones those who have notable vision issues, it’s definitely something we’re discussing with them right off the bat and in some cases when that’s the primary impairment and there aren’t any other say motor or stroke issues, there are many patients who end up being discharged home if they have supports” (male provider, acute)*

*“Yeah. I think … we screen…fairly well … in my opinion, we’re only starting baby steps at treatment. So, we screen well but…we’re … just starting to figure out how to treat” (female provider, tertiary)*

#### Living in the real world

ESD team providers spoke about the value of completing rehabilitation activities in home and community settings as they allowed the survivor to gain real-world compensatory experience. In community care settings, providers valued opportunities that allowed survivors to practice real-world skills in a safe and supervised context.

Survivors spoke about functioning with post-stroke VI and living in the real world in terms of compensation versus full recovery, driving, and transportation. While compensation was desired, some survivors felt that providers could address the possibility of visual recovery instead of focusing just on compensation strategies. Some survivors did not appreciate wholly negative prognoses with minimal chances of recovery. Those that spoke about benefitting more from the compensatory training tended to be older and seemed more open to living with a VI; their younger counterparts found it challenging to accept.

Other survivors emphasized that they were still able to do many things, such as complete household chores, prepare food, and exercise. Driving was emphasized as an essential part of daily living that was lost due to post-stroke VI and had a major impact on survivors’ sense of independence. This lost independence motivated survivors to seek rehabilitation. Several survivors felt that their vision improved over time and were in a rush to drive again; however, getting their licenses back was a “nightmare” as they needed to meet certain criteria and pass multiple tests.

“… that was one of many things that he told me that I would … never do, and he didn’t seem like he was … willing to give me … any direction” (female survivor, community)

*… so a lot of them [are] requesting training let’s say how to peel a vegetable, how to peel a carrot and how to cut a vegetable without hurting their fingers and also cooking is another big thing and when it comes to those who have really lost their vision, [we] make them turn on the stove. … [W]e do have patients that cause fire because they don’t really realize they … put a [wood] cutting board … on the stove and they turn it on and they cannot see it…” (female provider, community)*

“I still do my like … everyday things. House cleaning, cooking, baking … [nothing is] really affected [other] tha[n] I can’t drive anywhere” (female survivor, community)

## Discussion

Study results demonstrate the barriers and facilitators to fully supporting stroke survivors with VI across the care continuum. Challenges included barriers to access of appropriate services and providers; lack of available resources; as well as inconsistent collaboration and communication amongst multidisciplinary providers. Facilitators included early diagnosis and screening; provision of resources; clear referral pathways; and, vision rehabilitation that empowered survivors for real-life settings that were realistic and optimistic.

Both stroke survivors and providers discussed the prohibitive costs and associated lack of visual rehabilitation resources, which reduced accessibility and discouraged providers from offering these services. A pilot study surveyed 30 Canadian stroke survivors with VI and sent a questionnaire to ophthalmologists (*n* = 26), optometrists (*n* = 25), and opticians (*n* = 10) [21]. This study found that optometrists were the primary providers of visual rehabilitation such as conducting clinical assessments and dispensing visual aids, but the aids were deemed expensive by providers [21]. Private visual rehabilitation provision was deemed fiscally unsustainable [21]. Variation between provinces and between urban and rural areas affected the availability of funding for services and aids [21]. In the UK, a qualitative study with stroke survivors with VI (*n* = 35) focused on the manifestations of the condition versus interactions with the health system; but, the study did find that consistent challenges for stroke survivors were consistent lack of support and provision of information [22]. These findings complement our study and highlight the need for the visual rehabilitation funding model and scopes of practice of providers to be revisited to ensure balance and efficiency between the system and survivors. Our studies collectively highlight the importance of educating and empowering stroke survivors. Collaborations with hospitals should be explored to potentially reduce overhead costs for, and improve physical accessibility to, community-based vision providers.

Interprofessional communication challenges were seen as a barrier impeding access to, and quality of, visual rehabilitation. Some survivors felt that their care journey was delayed due to poor communication between providers. While providers reported that intra-team communication was strong, inter-team communication was sometimes lacking. Lam and Leat (2013) conducted a scoping review (*n* = 14 articles) on the barriers preventing individuals with post-stroke VI from seeking care; miscommunication between professionals was an identified access barrier to vision rehabilitation (*n* = 7 studies) [23]. A survey of Canadian optometrists (*n* = 459) revealed that while these professionals commonly referred to the national community organization for those with VI or blindness, only 10.7% of respondents received a written report after the referral [13]. These studies echo our findings and highlight the need for improved written and verbal communication between various professions and teams.

Referral pathways were discussed as a barrier to visual rehabilitation. Some providers noted that often, they did not know where or when to refer a patient and lacked clarity on other providers’ scopes of practice. This lack of clarity could lead to a lack of standardization and inequitable access for survivors. Latham et al. (2017) noted that at minimum, providers play the important role of knowing when to refer patients to low vision services and understanding the referral processes [24]. Clarifying referral pathways and processes for providers involved in the care of survivors with VI is therefore necessary.

The study had limitations. First, we interviewed less stroke survivors than providers, which may have skewed the data towards provider perspectives. However, most of the patient interviews lasted longer than the provider interviews which allowed for greater depth of insight and analysis. Second, selection bias may have arisen as there may have been commonalities between individuals who did not consent to be contacted during the recruitment process. However, we heard from providers that the majority of those approached about the study were keen to participate because they recognized the importance of the work. Finally, we did not interview any survivors immediately following their acute care experience due to public-health restrictions related to the global pandemic. Any different experiences in acute care may not have been captured therefore limiting the transferability of results to this area. However, many survivors had an acute experience which may help mitigate this limitation.

## Conclusion

We sought to identify and describe the barriers and facilitators experienced by survivors with post-stroke VI across the care continuum. A provincial group translated these qualitative findings of this paper into a cross-sectional, provincial survey with the aim of hearing from more survivors and providers across the province, and to prioritize the identified gaps for action. The findings from both studies will facilitate the development of a provincial low vision care pathway for stroke survivors that will aim to address gaps and challenges experienced and prioritized by the community. Future research will focus on evaluating the effectiveness of this care pathway from both a survivor and provider perspective in order to continue monitoring and improving post-stroke VI care provision in Alberta.

## Data Availability

The datasets generated and/or analysed during the current study are not publicly available due to their qualitative nature, but select, de-identified quotes are available from the corresponding author on reasonable request.

## Abbreviations

ESD: Early Supported Discharge
VI: Visual impairment
